# Comparative study between lateral extra-articular tenodesis versus anterolateral ligament reconstruction in combination with anterior cruciate ligament reconstruction

**DOI:** 10.1186/s13018-025-06561-x

**Published:** 2026-01-17

**Authors:** Hossam El-Azab, Omar Abdelkareem, Abdel Rahman Hafez, Moustafa Elsayed, Mohamed Ali

**Affiliations:** Department of Orthopaedics and Traumatology, Sohag Faculty of Medicine, Sohag University, Sohag, Egypt

## Abstract

**Background:**

Residual laxity after anterior cruciate ligament reconstruction (ACL-R) is found in some cases. Nowadays, anterolateral augmentation procedures are performed to prevent this laxity.

**Materials and methods:**

A prospective randomized comparative clinical study took place from January 2021 to November 2022. The study included 48 patients after exclusion of losses to follow-up, who acquired ACL-R combined with either lateral extraarticular tenodesis (LET) in 25 or anterolateral reconstruction (ALL) in 23 patients. The patients were evaluated with the Tegner-Lysholm score, and follow-up was for 26 ± 7 months. Also, laxity tests, pivot shift and Lachman preoperatively and postoperatively were evaluated. The failure rate was assessed.

**Results:**

Tegner-Lysholm score improved significantly from 51.28 preoperatively to 93.76 points two years postoperatively in the LET-group and from 54.55 preoperatively to 94.23 points two years postoperatively in the ALL-group (*P*-value < 0.0001 in both groups). There was no significant difference between values of the Tegner-Lysholm score for the two groups at two-year follow-up (*P*-value = 0.24). Also, there was a significant reduction in the number of patients with positive pivot shift and Lachman tests in each group (*P* value < 0.0001 in both groups). The failure rate was 8% for LET and 8.7% for ALL (*P*-value = 0.46). Revisions were done from the contralateral side within the follow-up period.

**Conclusions:**

The clinical outcome and stability after anterior cruciate ligament reconstruction combined with either lateral extraarticular tenodesis or anterolateral ligament reconstruction improved significantly after two years. However, no significant difference between them was found after two years. Both groups had comparable failure rates. However, the intraoperative and postoperative findings support anterolateral ligament reconstruction.

**Level of evidence:**

Level 1, prospective randomized comparative study.

**Trial registration:**

Clinical trial registry: NCT06222814. Registered at 01/01/2024, retrospectively.

**Key points:**

Comparative study between extraaricular tenodesis and anterolateral reconstruction combined with ACL-R., Clinical outcome after ACL reconstruction combination. Anterolateral Ligament Reconstruction or extraaricular tenodesis, rotatory and anterior laxity knee, lateral stabilization of the knee.

## Introduction

Residual instability of the knee joint remains a major concern after successful anterior cruciate ligament reconstructions (ACL-R). It accounts for more than 25% of patients [1]. Those patients complain of an inability to return to their previous level of sports [2–4].

Recently, considerable attention has been given to residual instability of the knee as a major issue regarding when patient can return to sports after ACL-R [5]. This instability should be addressed at the time of ACL surgery to improve patient outcomes [6]. Addressing this instability can be done with an anterolateral augmentation. The anterolateral augmentation procedures are either lateral extraarticular tenodesis (LET) or anterolateral ligament reconstruction (ALL-R) techniques [6–8].

There are many techniques for LET from the iliotibial band (ITB) for the femoral side attachment, either an interference screw, staple, a suture anchor [9] or only a high-resistance suture [10].

Also, many modifications in ALL reconstruction techniques have been reported, all of which suggest varying femoral fixation points [11]. Also, ALL grafts are tendons, either from the hamstring or the peroneus longus [12].

Despite the biomechanical evidence suggesting that LET or ALL combined with ACL-R have a better outcome regarding anterolateral instability compared with isolated ACLRs [13–17], the clinical differences of these procedures are still debatable.

The comparison of either additional procedure to ACL reconstruction was the objective of this study. The hypothesis was that tendon reconstruction in ALL is better than the iliotibial tract tenodesis in the LET technique in combination with ACL-R.

## Materials and methods

This study was conducted to determine which of the two maneuvers is better to augment ACL-R, either with ALL or LET procedures, to improve the outcome. The study was approved by the local ethics committee with the approval number 395/2020. Informed consent was signed by every patient before participation. A prospective randomized comparative clinical study with a parallel group design took place from January 2021 to November 2022. Sixty patients with an ACL tear and highly positive pivot shift tests (grade II or III) were operated on, and follow-up was for 26 ± 7 months. Randomization was performed at patient admission to allocate the patients into two groups, 50% each: ACL-R with LET and ACL-R with ALL.

Patient demographic data are enumerated in Table 1.

**Table 1:** Patient demographic data (N. 25; LET and No. 23 ALL groups)

Parameter	LET group	ALL group
Mean age at surgery (years) (SD)	26 (**± **6.7)	24 (**± **5.4)
Male/female	21/4	19/4
Athletes	21	18
Professional	7	5
Side (right/left)	17/8	16/7
*Mechanism of injury* Sport injury Social Motor car accident	15 6 4	15 5 3
Interval between injury and surgery (months) [18]	8 (**± **3.7)	7 (**± **5.7)
*Meniscal surgery* Meniscal-repair No (%) Menisectomy No (%)	4 (16%) 7 (28%)	4 (17.4%) 6 (26%)

N: Number, SD: standard deviation

Patients were collected from the sports medicine clinic, examined clinically, and investigated with MRI.

### Inclusion criteria

- ACL rupture detected by clinical examination and Magnetic Resonance Imaging (MRI).
- Skeletally mature patient (Age 18–35 years).
- High positive pivot shift test of grade II or III.

### Exclusion criteria

1. Revisions or ACL rupture in the opposite knee.
2. Multiple Ligament injuries.
3. Articular cartilage defect requiring treatment; Outerbridge more than grade II.
4. Malalignment, more than three degrees (varus or valgus)
5. General hyperlaxity.

### Surgical technique

Anatomic ACL reconstruction was performed in a standardized fashion by the same surgeon in all cases. Choice of procedure was made on admission to the department before surgery, either to do ACL-R combined with LET or ALL in a 1:1 ratio with complete randomization.

### ACL-R with LET

ACL-R was performed in the standard fashion using a triple hamstring graft [19, 20]. Semitendonosus-Gracilis (STG) tendon graft (6 strands). Femoral and tibial fixations were performed with bioabsorbable interference screws (Smith & Nephew Endoscopy). Then, the modified Lamaire procedure was performed according to.

**Fig. 1:**
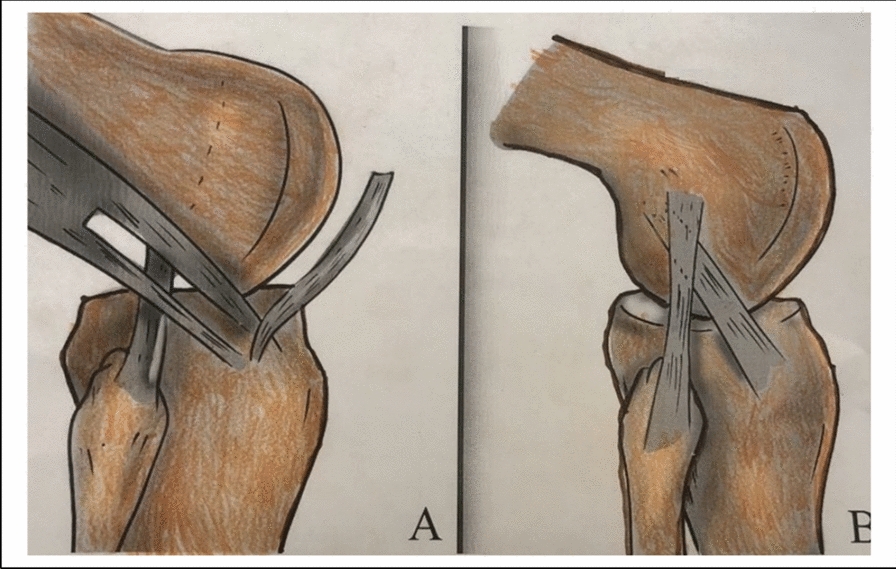
Illustration showing LET technique

**Fig. 2:**
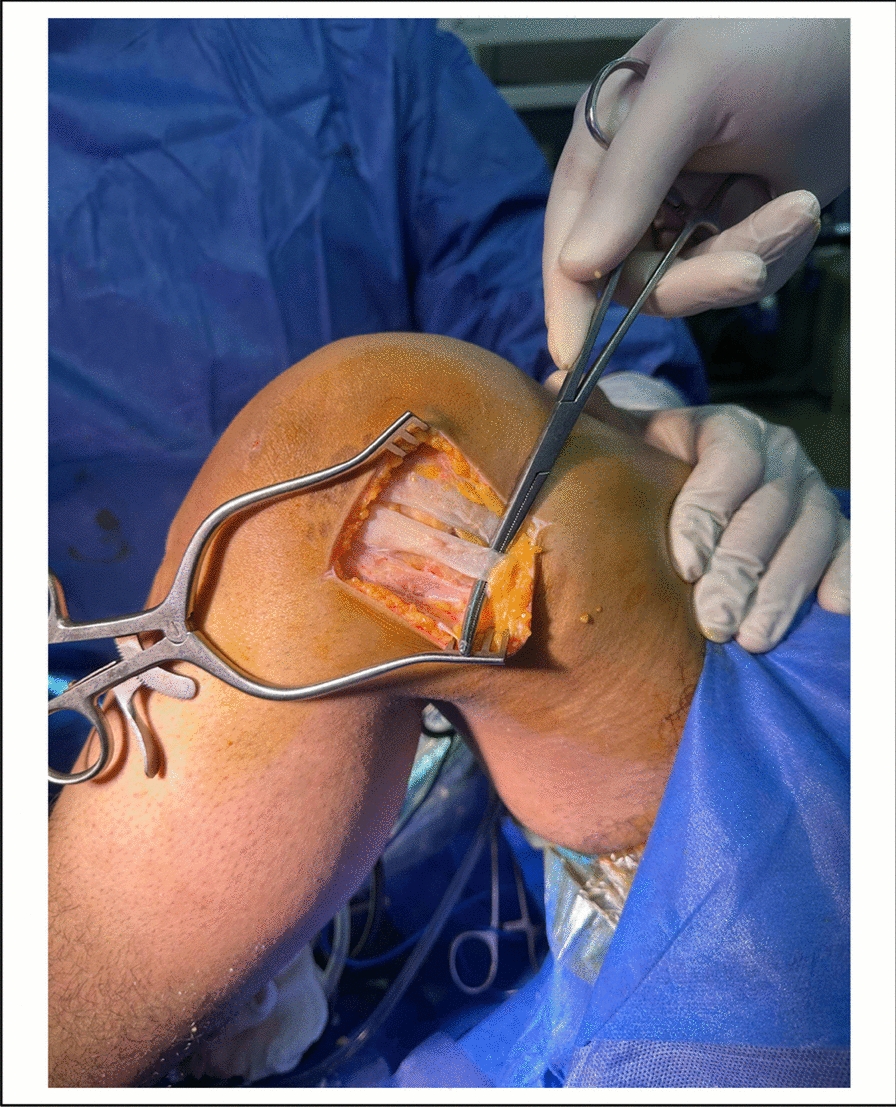
Intra-operative photo, present strand of LET in the ITB before cutting

**Fig. 3:**
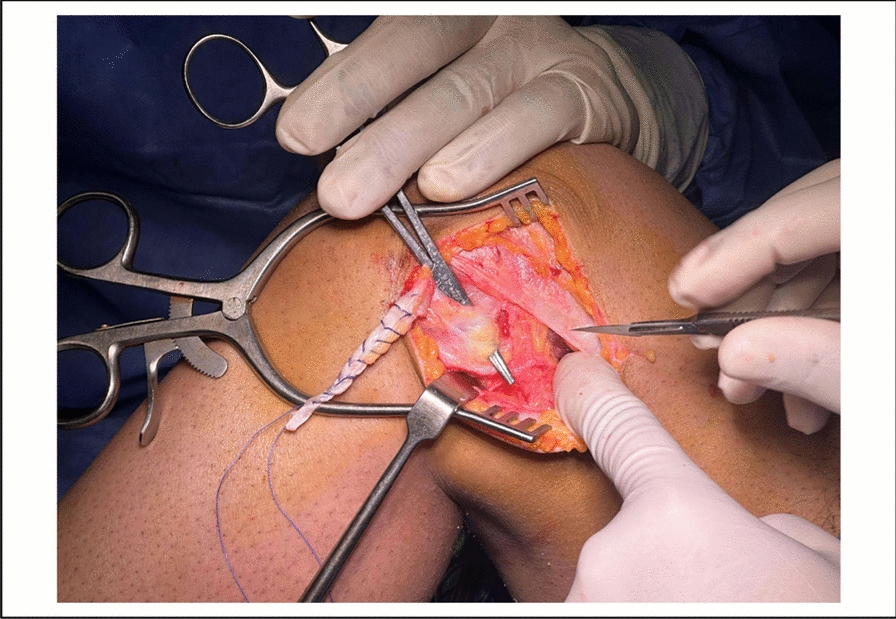
Intra-operative photo showing how the prepared ITB graft passed under LCL

**Fig. 4:**
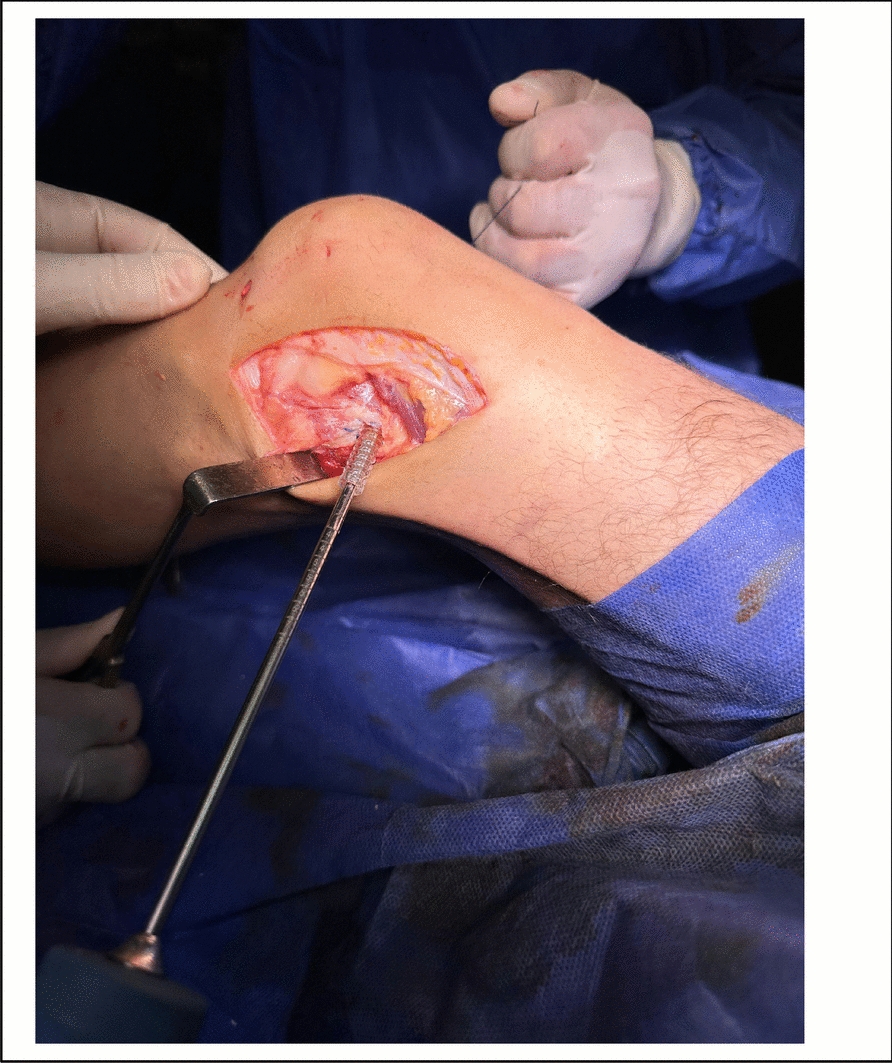
Intra-operative photo showing fixation of LET graft in the femoral tunnel by an interreference screw

Getgood et al. [21] (Figs. 1, 2, 3 and 4). The iliotibial band graft diameter was measured. Fixation at the distal femur was done with the knee held at 60° of flexion and neutral rotation of the tibia.

### ACL-R with ALL

**Fig. 5:**
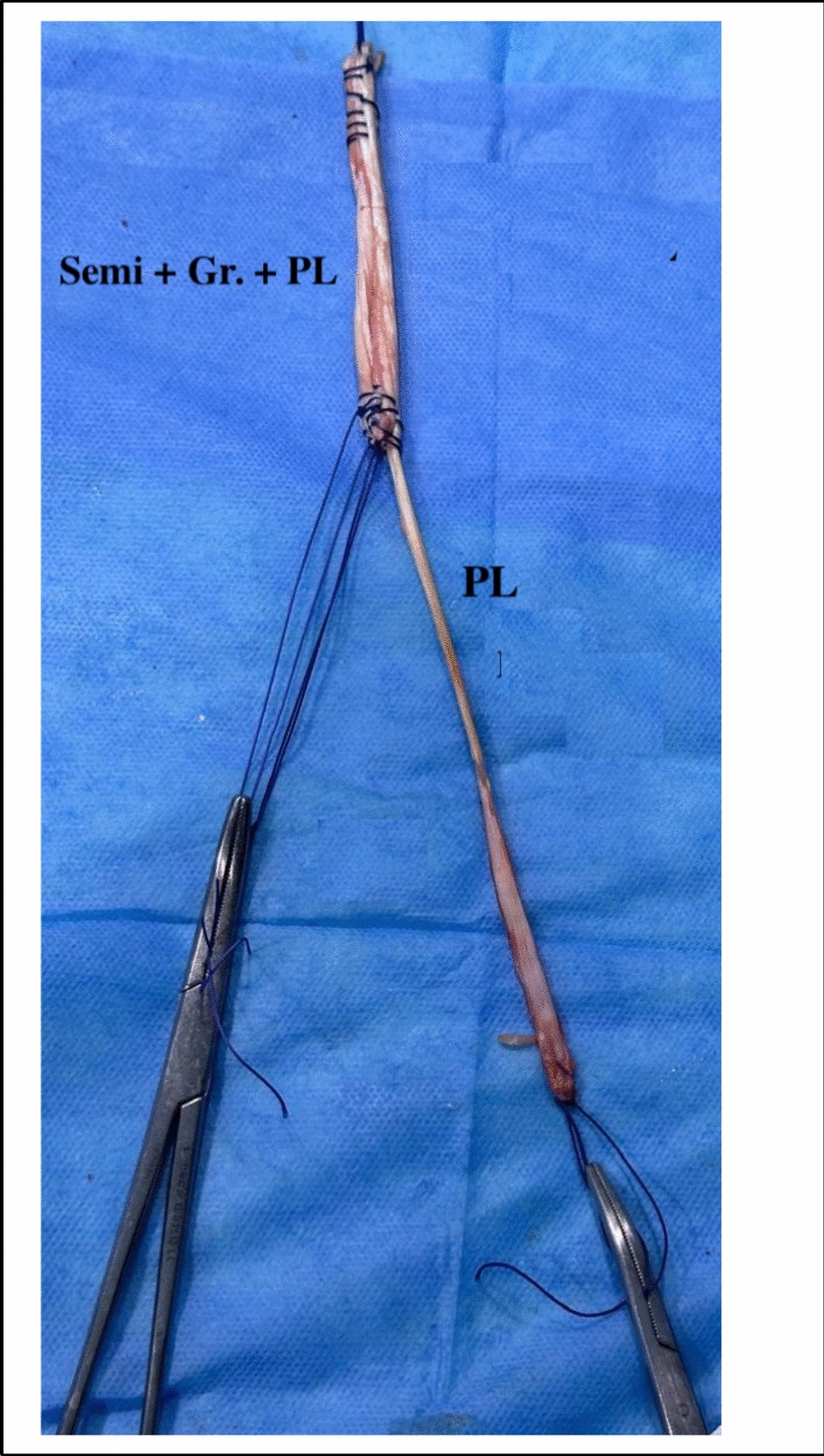
Intra-operative photo showing the quintuple (5 strands, 4 hamstrings + one peroneus longus) graft for ALL

**Fig. 6:**
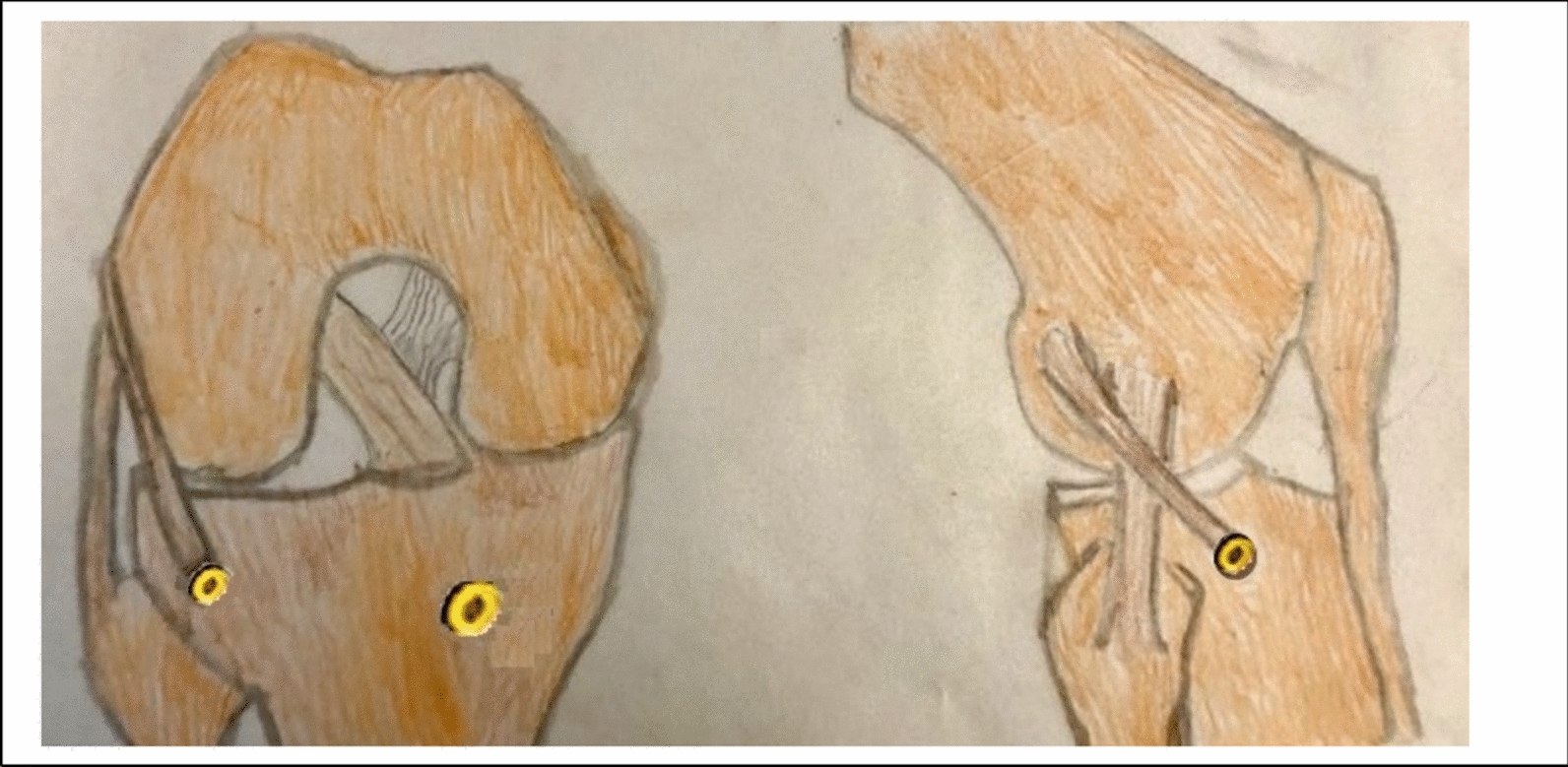
Illustration of ALL technique in the knee,

**Fig. 7:**
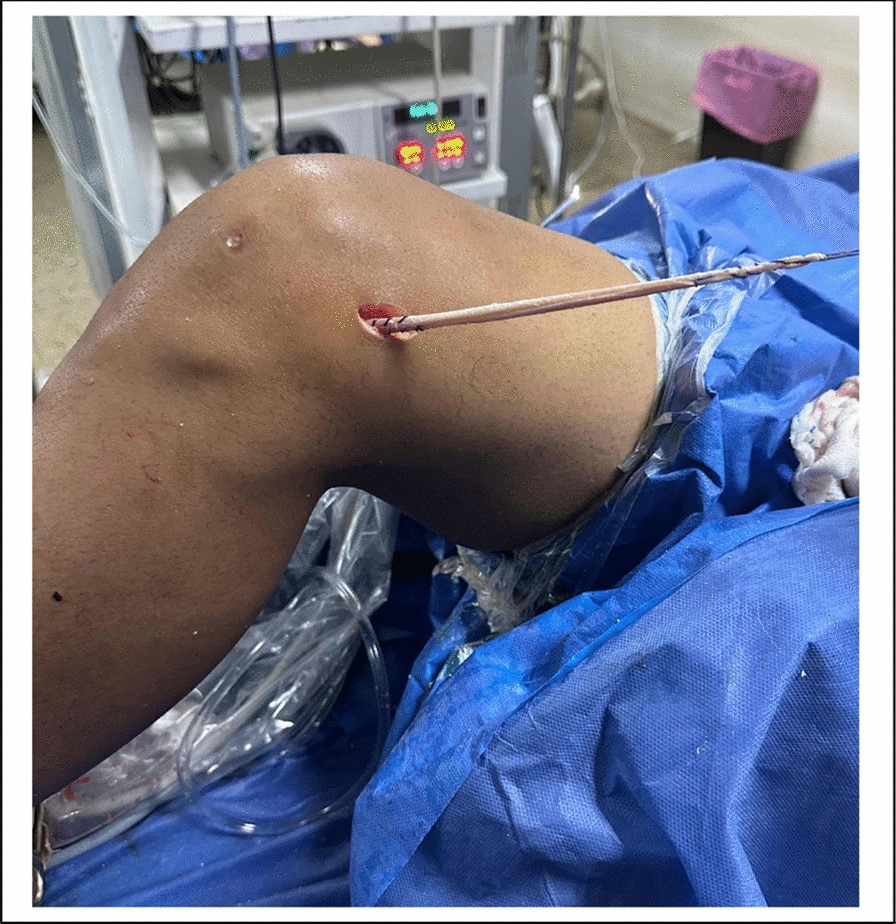
Intra-operative photo showing the single strand peroneus longus graft emerging at femoral opening

**Fig. 8:**
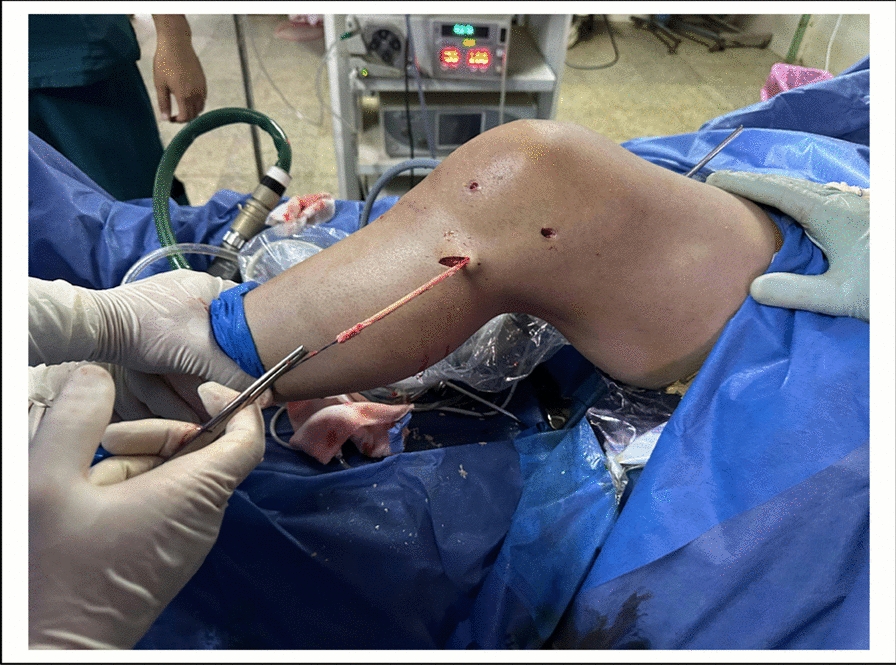
Intra-operative photo showing the single strand peroneus longus graft routed downwards to the proximal tibia, emerging at proximal tibia

ACL-R was performed in the standard fashion according to de Oliveira et al. [22] STG in quadruple strands [20] plus the peroneus longus tendon in a single strand (total 5 strands) (Fig. 5). ACL-graft and peroneus longus tendon graft diameters were measured. Femoral and tibial fixations were performed with bioabsorbable interference screws (Smith & Nephew Endoscopy). Fixation of the ALL graft at the proximal tibia with the knee held at 30° of flexion and mild valgus ( Figs. 6, 7 and 8).

### Rehabilitation

All patients, whatever the group, underwent identical postoperative physiotherapy rehabilitation unless a meniscal repair was concomitantly done. Meniscal repair was performed in eight patients (four in the LET group and four in the ALL group). Attention was given to meniscus repair in the first six weeks [23], then they continued the rehabilitation program as the other patients.

Rehabilitation was tailored for each patient, and postoperative visits were frequent for the professional players to guide them. It aimed at preventing reinjury and enabling return to sports [24].

Physical rehabilitation for ACL-R with augmentation was carried out as reported by El-Azab et al. [25].

## Outcome measures

***The primary outcome measure*** was the Tegner-Lysholm Knee Score (TLS) [26], designed to evaluate the clinical outcomes of knee ligament surgery, particularly for symptoms related to instability; therefore, it was suitable.

TLS was originally published in 1982 and later modified in 1985 [26] and is a patient-reported questionnaire consisting of pain (25 points), instability (25 points), locking (15 points), swelling (10 points), limp (5 points), stair climbing (10 points), squatting (5 points), and the need for support (5 points). Scores range from 0 (maximum instability) to 100 (no instability). The examination was done both preoperatively (preop.) and at two-year postoperatively (postop.).

**S*econdary outcome measures*** were the pivot shift test and its grading according to Jakop [27], the Lachman test and its grading according to Gurtler [28], and complications (Table 6).

Follow-up examinations were done in the outpatient clinic and planned for 2 weeks, 6 weeks, 6 months, 1 year, and 2 years postoperatively. Professional athletes were followed as the other patients, but at nine months, follow-up was weekly during their return to play to guide them.

In the last follow-up, patients filled out TLS helped by the clinician, who explained and translated the score. To reduce bias, the clinician was blinded to the clinical data, and the patients’ knees were covered with a bandage to hide individual groups.

### Statistical analysis

Analysis of data was done with SPSS software (Version 25; SPSS Inc, Chicago, Illinois, USA). Descriptive statistics for variables such as age, sex, duration of symptoms, and concomitant meniscus lesion were calculated. The mean was calculated for the TLS score. Paired student tests (*P*-value) were used to compare TLS before and two years after the operation and between individual groups at the final follow-up. A *p*-value of less than or equal to 0.05 was considered statistically significant.

In this study, we used a convenience sample, where all cases admitted to the hospital in the study period, between January 2021 and November 2022, who agreed to the inclusion and exclusion criteria and agreed to participate in the study, were included. So, power analysis was not performed.

## Result

Sixty consecutive patients were operated on in both groups. There were 49 men and 11 women, 38 right and 22 left knees. (Table 1). Five patients from the LET group and seven patients from the ALL group were lost to follow-up. Finally, 48 patients (80%) or knees, as there were no bilateral cases, participated in the study. Further, at the final follow-up, two patients from each group were excluded due to ACL-R failure. Therefore, there were 23 patients in the LET group and 21 patients in the ALL group.

TLS in both LET and ALL groups showed significant improvement after two years (Tables 2 and 3). However, the improvement at two-year follow-up between both groups was nonsignificant (*P*-value = 0.24) (Table 4).

**Table 2:** Tegner-Lyshom score for the group of LET, preop. and postop. (N. 25 at surgery and 23 at two-year follow-up) (Fig. 9)

Tegner-Lyshom score	Tegner-Lyshom score preop	Tegner-Lyshom score Postop	P-value
Limp (5 points)	1.88 ± 3.3	4.36 ± 0.2	< 0.00001
Support (5 points)	4.04 ± 2.7	4.28 ± 0.7	0.004638
Locking (15 points)	8.12 ± 7.4	14.2 ± 0.7	< 0.00001
Instability (25 points)	8.8 ± 6.5	23.8 ± 0.8	< 0.00001
Pain (25 points)	14.4 ± 5.3	24.0 ± 0.8	< 0.00001
Swelling (10 points)	5.68 ± 2.4	8.72 ± 0.5	< 0.00001
Stair-climbing (10 points)	4.96 ± 3.6	9.04 ± 0.5	< 0.00001
Squatting (5 points)	3.4 ± 2.5	4.36 ± 0.6	< 0.00001
Total (100 points)	51.28 ± 19.62	93.76 ± 15.33	< 0.00001

Preop: Preoperative, Postop: Postoperative

**Table 3:** Tegner-Lyshom score for the group of ALL, preop. and postop. (N. 23 at surgery and 21 at two-year follow-up) (Fig. 9)

Tegner-Lyshom score	Tegner-Lyshom score preop	Tegner-Lyshom score postop	P-value
Limp (5 points)	1.91 ± 2.4	4.26 ± 0.4	< 0 00001
Support (5 points)	3.08 ± 1.7	4.36 ± 0.6	0.009597
Locking (15 points)	7.86 ± 6.5	13.91 ± 0.8	< 0.00001
Instability (25 points)	6.89 ± 7.7	23.8 ± 1.2	< 0.00001
Pain (25 points)	13.08 ± 9.8	24.13 ± 0.6	< 0.00001
Swelling (10 points)	5.82 ± 4.5	9.13 ± 0.5	< 0.00001
Stair-climbing (10 points)	5.39 ± 1.8	9.13 ± 0.6	< 0.00001
Squatting (5 points)	2.52 ± 3.4	4.6 ± 0.3	< 0.00001
Total (100 points)	54.55 ± 19.5	94.23 ± 9.6	< 0 .00001

Preop: Preoperative, Postop: Postoperative

**Table 4:** Comparison of Tegner-Lyshom score between the group of LET (N. 23) and the group of ALL (N. 21) at two-year follow-up

Tegner-Lyshom score	Tegner-Lyshom score LET group	Tegner-Lyshom score ALL group	*P*-value
Limp (5 points)	4.36 ± 0.2	4.26 ± 0.4	0.217488
Support (5 points)	4.28 ± 0.7	4.36 ± 0.6	0.953224
Locking (15 points)	14.2 ± 0.7	13.91 ± 0.8	0.309886
Instability (25 points)	23.8 ± 0.8	23.8 ± 1.2	0.528064
Pain (25 points)	24.0 ± 0.8	24.13 ± 0.6	0.420886
Swelling (10 points)	8.72 ± 0.5	9.13 ± 0.5	0 .217488
Stair-climbing (10 points)	9.04 ± 0.5	9.13 ± 0.6	0.628064
Squatting (5 points)	4.36 ± 0.6	4.6 ± 0.3	0 .689878
Total (100 points)	93.76 ± 15.33	94.23 ± 9.6	0 .249659

Patients experienced a satisfactory feeling of stability at final follow-up, despite the result of the pivot shift test found at physical examination (Table 5).

**Table 5:** Pivot shift and Lachman tests of patient’s knees before the operation and at final follow up

Pivot shift test	Group of LET		*P* value	Group of ALL		*P* value	*P* value
	Pre	Post	pre & post	Pre	Post	pre & post	bet. LET & ALL at final follow up
Negative	0	20	< 0.0001	0	19	< 0.0001	0.13657
Positive GI	0	3		0	1		
Positive GII	10	0		9	1		
Positive GIII	15	0		14	0		
*Lachman test*							
Negative	0	21	< 0.0001	0	20	< 0.0001	0.21537
Positive GI	0	2		0	1		
Positive GII	11	0		8	0		
Positive GIII	14	0		15	0		

The difference after two years for each group together with the difference between both groups (positive tests) at two years follow-up were recorded. (N. preop. LET 25, ALL 23 but N. at final follow up. LET 23 and ALL 21)

Preop: Preoperative, Postop: Postoperative

There were five patients at final follow-up with a positive pivot shift test (three patients in the LET group (13%), grade I, and two patients in the ALL group (9.5%), grade I and II). Those five patients underwent MRI, which showed an intact ACL graft. The Lachman test was positive in three patients in both groups, grade I in two patients (8.7%) in the LET group and one patient (4.7%) in the ALL group. The differences between both groups regarding pivot shift and Lachman tests at final follow-up were nonsignificant (*P*-value was 0.13, 0.21, respectively).

Different parameters were measured between the two methods and recorded (Table 6).

**Table 6:** Characters of LET and ALL groups (LET group N. 25 at surgery and 23 at two-year follow-up, ALL group N. 23 at surgery and 21 at two-year follow-up)

Character, parameter	ACL-R with LET from ITB	ACL-R with ALL From PL	*P* value
Anatomical	Non-anatomical	Anatomical	
Invasiveness (Fig. 10)	More invasive	Less invasive	
Graft Length changes, histology	Isometric as a non-stretchable structure contain no elastic fibers, elongated	Non-isometric contains elastic fibers stretchable, no elongation	
Graft harvesting time, min	13.48 ± 4.6	5.0 ± 3.5	0.00028
Mean graft diameter mm	4.1 ± 2.7	6.54 ± 2.93	0.03391
Mean incisions length, cm	8 ± 5	3.5 ± 3.5	0.00124
Return to sports at 9 months (N.) Return to previous sport level at 11 ± 4 mo. (N.)	21 17	18 15	0.309886 0.421147
Complications Infection, (N.) Lateral knee pain at 6 weeks (N.) at 6 months (N.) ACL Failure	2 11 4 2/25 (8%)	1 7 0 2/23 (8.7%)	0.305319 0.158641 0.023095 0.466202

## Discussion

The most important findings in this study were the significant improvement of clinical scores after ACL surgery combined with an augmentation procedure and no residual instability. However, this improvement did not differ significantly between the LET and ALL groups after two years.

TLS for the LET group was improved significantly at follow-up. This corresponds to Goncharov et al. [29], who found TLS improved significantly in medium-term follow-up after LET.

TLS for the ALL group was improved significantly at follow-up. This corresponds to Rosenstiel et al. [30], who found Lysholm scores improved significantly at follow-up after 3.9 years [31].

In the current study, TLS between both groups was not significantly different at follow-up (*P*-value = 0.24). In contrast to Helito et al. [32] in their retrospective study, who found a significant improvement of Lysholm scores [31] of the ALL group than the LET group (*P*-value = 0.047).

There is a significant reduction in positive pivot shift and Lachman tests between preoperative and two-year follow-up for both groups (Table 5). This confirmed the efficacy of both procedures in reducing hyperlaxity suggested in biomechanical studies [9, 13, 14]. However, the differences in pivot shift and Lachman tests between both groups at two-year follow-up were nonsignificant. This result corresponds to the retrospective study by Helito et al. [32], who found that the difference in the pivot shift test and KT-1000 examination (instrumental Lachman) between both groups was nonsignificant.

Lateral-side knee pain was described more by LET than ALL patients at 6 weeks, then only by LET patients at 6 months (Table 6). This result is similar to Helito et al. [32], who found lateral side knee pain lasted for 4.3 ± 2.1 months after LET and for 2.2 ± 1.3 months after ALL. The difference was significant (*P* value < 0.00001).

The incidence of ACL failure in this study was 8% for the LET and 8.7% for the ALL groups, and the difference was nonsignificant (*P* value = 0.46) (Table 6). This corresponds to Helito et al. [32], who found a failure rate of 2.2% for the LET group and 7.3% for the ALL group, and the difference was nonsignificant (*P*-value = 0.34). Revisions of failure here enabled a second look after the combined procedure, and were not mentioned in the literature before. Revision, also of augmentation, was done, either from the same side in the case of LET or the contralateral side in the case of ALL.

No biomechanical superiority of either ALL or LET combined with ACL-R was found [33]. However, several comparative biomechanical studies between LET and ALL combined with ACL-R suggested that LET restricts rotatory instability more than ALL-R [14]. Also, in other biomechanical studies [9, 13, 14], the incidence of overconstraint of the knee is a permanent sequel in all literature after LET, but is controversial after ALL.

Our hypothesis was not confirmed regarding the clinical outcome; ALL was not better than LET after two years of follow-up. However, intraoperative observation and findings in follow-up can help us make a decision.

**Fig. 9:**
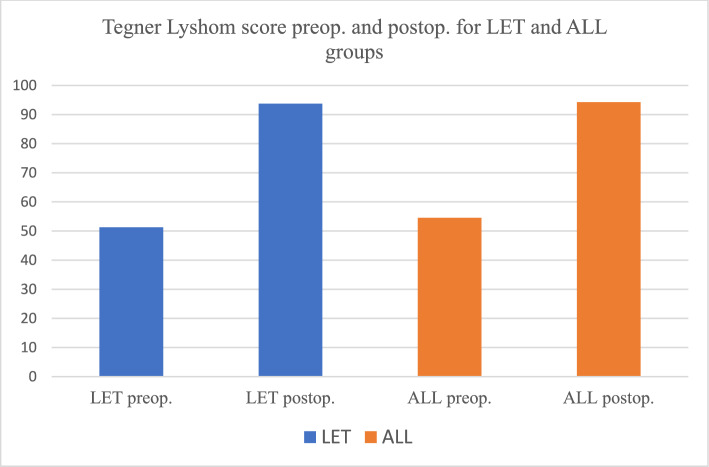
Graph showing LET and ALL change between preop. and postop

**Fig. 10:**
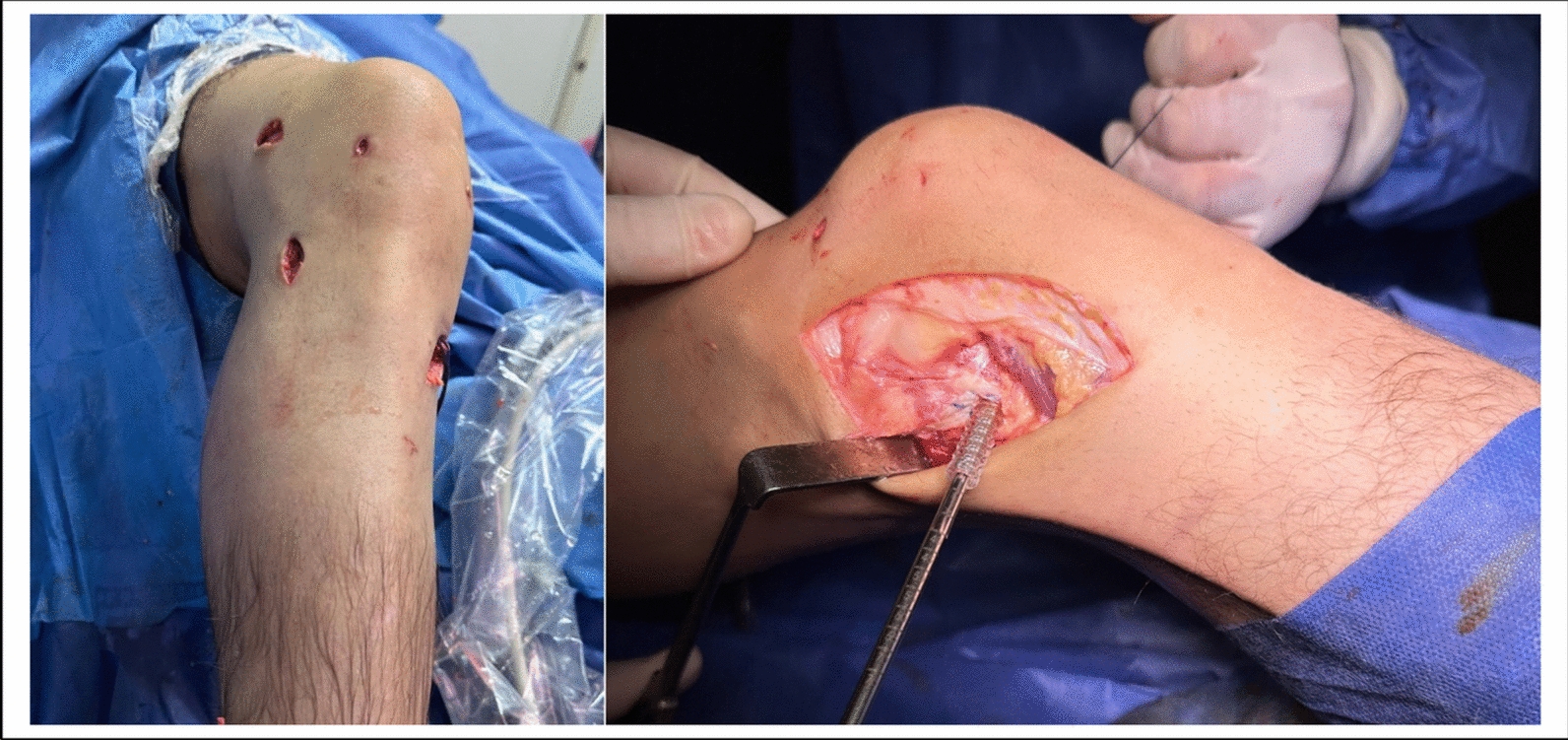
Intra-operative photo showing comparison of skin incisions of LET and ALL

“ àla carte approach“ was suggested by Shybut [34], who recommended that when pivoting, action is needed, LET is best performed, but when lesser demands are needed, ALL is best performed. We don’t agree; our argument is the expected deterioration of the function after LET in long-term follow-up. Also, Maffulli et al. [35], in their systematic review, concluded that [35]. LET was associated with a higher reoperation rate than ALL. So, it is not recommended.“ àla carte approach “ was suggested by Shybut [34], who recommended that when pivoting, action is needed, LET is best performed, but when lesser demands are needed, ALL is best performed. We don’t agree; our argument is the expected deterioration of the function after LET in long-term follow-up. Also, Maffulli et al. [35], in their systematic review, reported greater clinical outcomes for LET. However, in the long term, we expected deterioration. In addition, they concluded that [35]. LET was associated with a higher reoperation rate than ALL. So, it is not recommended (Figs. 9, 10).

### Native anterolateral ligament (ALL) (Fig. 11).

**Fig. 11:**
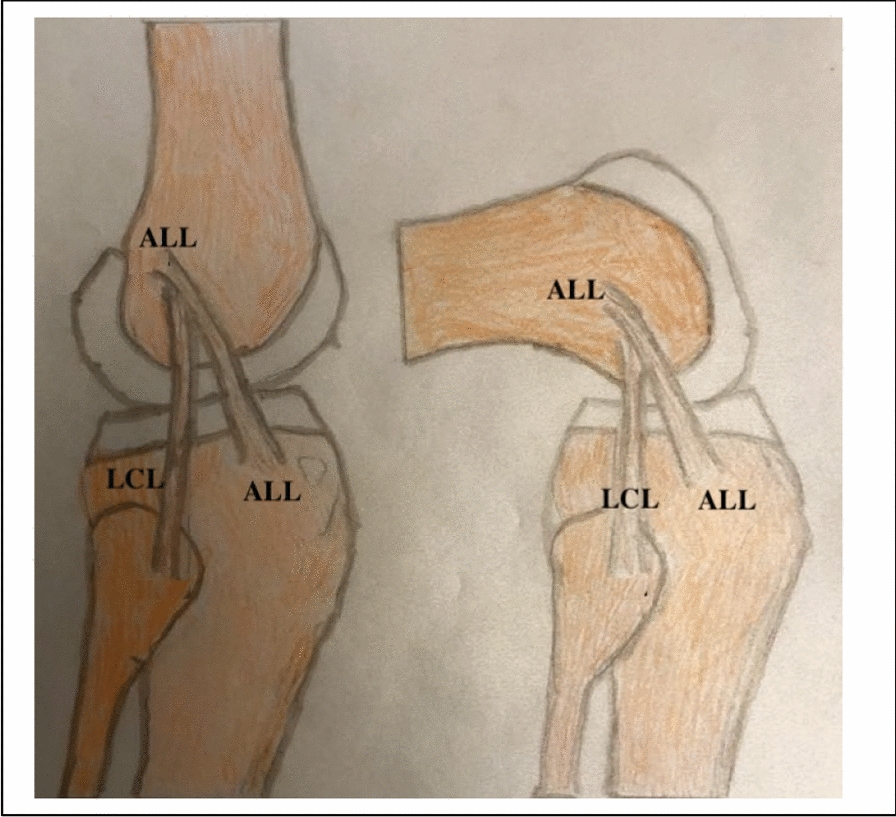
Illustration of native ALL and its attachments

The structure was first described by Segond in 1879 and redescribed by Claes in 2013 [36]. Ahn et al. [37] reported, the constant femoral attachment of the ALL as posterior and proximal to the lateral femoral epicondyle (LFE). ALL runs obliquely along the anterolateral aspect of the knee. Its attachment distally on the anterolateral tibia was situated midway between Gerdy’s tubercle and the fibular head. The histological findings of the ALL demonstrate a separate ligamentous structure, which was clearly distinguished from the anterolateral knee joint capsule and the ITB [38].

Quantitative analysis of ALL was reported by Helito [39],, mean length of 41.5 ± 6.7 mm in 90° of flexion and 38.5 ± 6.1 mm in full extension. The mean width was 8.3 ± 2.1 mm. ALL contains elastic fibers, which allow the ligaments to stretch to some extent. Elastin content has been reported to range from 0.25% to 10% of the tissue’s dry weight; so, it is stretchable during knee motion [40].

Biomechanical studies [33, 41] have shown that the native ALL functions as a secondary stabilizer to the ACL in resisting anterior tibial translation and internal tibial rotation. However. ALL has a minor role in the presence of an intact ACL. Several cadaveric studies [42, 43] demonstrated the ALL and investigated the role of the ALL on rotatory instability in ACL deficiencies by sequential sectioning of the ACL and ALL [44]. They found that the role of ALL in rotatory instability increased in ACL-deficient knees.

### Anterolateral ligament reconstruction (ALL) ( Figs. 5, 6, 7 and 8).

Several biomechanical studies demonstrated improved rotational instability and restoration of normal knee kinematics through ALL-R combined with ACL-R [11, 45].

It is advantageous to use the peroneus longus tendon here because it is sufficiently long while preserving the large-diameter status of the ACL graft. The large-diameter ACL graft decreases the incidence of failure [25, 46]. When hamstring tendons are used for ALL-R [47], this will be at the expense of ACL graft diameter.

ALL-R, using a tendon (hamstrings or peroneus longus tendons) that mainly contains collagen and elastin fibers [48]. So, its structure is suitable to reproduce the native ALL ligament.

ALL-R has separate attachment points close to the native ALL attachment sites of the femur and tibia (Fig. 9).

ALL-R is anatomically based and can be percutaneously performed with one or two small incisions on the femoral and tibial sides. ALL-R, when correctly fixed in full extension and neutral rotation, has been shown to restore normal knee kinematics and avoid overconstraint, as reported by Inderhaug et al. [41]. This makes a strong predilection for this procedure. Also, Getgood et al. [49] found with ALL-R, there was no incidence of overconstraint of the knee. Neri et al. [50] reported that ALL-R did not elevate the lateral femorotibial pressure. In contrast, an anatomic ALL-R in conjunction with ACL-R, was reported by Engebretsen et al. [51], led to a significant overconstraint of the knee. DePhillipo et al. [11] reported overconstraints of internal rotation using the posterior/proximal ALL femoral attachment point, but no overconstraints using the anterior/distal ALL femoral attachment point.

### Lateral extraarticular tenodesis (LET) (Figs. 1, 2, 3 and 4).

LET is an augmentation procedure for providing added stability to the lateral aspect of the knee and reducing anterolateral knee instability [52]. It was performed with a strand of ITB fixed over the knee joint [21].

Histologically, the ITB consists mainly of collagen fibers, as confirmed by Kittl et al. [53]. There is a small amount of elastic fibers amongst the layers of collagen, which allow it to be slightly elastic, helping it act as a spring. However, this does not give it the ability to stretch like a muscle. So, in various knee motions with length changes, the graft may be stretched or elongated as it is isometric. Kernkamp [54] reported, an increase in the distance between the attachment points of 6% could lead to permanent graft elongation and eventual graft failure.

We theorize, LET subsequently may not restore the native kinematic state of the knee in the long term because LET often tends to elongate over time, with the return of anterolateral rotatory instability.

Additionally, there is concern regarding the nonanatomic nature of LET procedures, the potential for overconstraining the knee, and restricted internal tibial rotation [14], which led to a decrease in their popularity. Neri et al. [50] and Inderhaug et al. [41] reported, early arthritis in the lateral compartment after the LET. This was confirmed by MRI analysis of the lateral compartment, where Firth et al. [55] found early changes in the articular cartilage of the lateral compartment after the LET.

There are some limitations of the study, including the study population being limited to 23 LET and 21 ALL patients. Also, no power analysis was performed, which may not be enough to detect a significant difference. About surgical technique, ACL reconstruction was performed differently between the two groups (triple hamstring graft in LET versus STG in quadruple strands plus peroneus longus tendon in a single strand in ALL), which may have biased the results. Although patients covered their knees with a bandage, the individual groups were not completely concealed, and the investigator still could differentiate. The lack of blinding leads to bias, affecting the overall result. The pivot shift assessment is based on the skills of the examiner, which could bias the results of rotational instability in this study. Regional differences in the physiotherapy program between patients were possible. However, despite our appreciation of the limitations of our investigation, we believe that the results of this study could be useful in the future development of prospective long-term comparative studies.

## Conclusions

The clinical outcome and stability after anterior cruciate ligament reconstruction combined with either lateral extraarticular tenodesis or anterolateral ligament reconstruction improved significantly after two years. However, no significant difference between them was found after two years. Both groups had comparable failure rates. However, the intraoperative and postoperative findings support anterolateral ligament reconstruction.

## Data Availability

The datasets used and/or analyzed during the current study are available from the corresponding author on request.

## References

[1] Maffulli N, Colombet P, Oliva F, Oliviero A. Combined anatomic reconstruction of the anterior cruciate and anterolateral ligaments, how i do it. Muscles, Ligaments Tendons J MLTJ. 2019;9(3):457–62.

[2] Kuroda R, Hoshino Y, Araki D, Nishizawa Y, Nagamune K, Matsumoto T, et al. Quantitative measurement of the pivot shift, reliability, and clinical applications. Knee Surg Sports Traumatol Arthrosc. 2012;20(4):686–91. 10.1007/s00167-011-1849-6. (**in eng**). 22210517 10.1007/s00167-011-1849-6

[3] Papalia R, Franceschi F, Tecame A, D’Adamio S, Maffulli N, Denaro V. Anterior cruciate ligament reconstruction and return to sport activity: postural control as the key to success. Int Orthop. 2015;39(3):527–34. 10.1007/s00264-014-2513-9. (**in eng**). 25192689 10.1007/s00264-014-2513-9

[4] Maffulli N, Osti L. ACL stability, function, and arthritis: what have we been missing? Orthopedics. 2013;36(2):90–2. 10.3928/01477447-20130122-02. (**in eng**). 23379658 10.3928/01477447-20130122-02

[5] Webster KE, Feller JA, Kimp AJ, Whitehead TS. Low rates of return to preinjury sport after bilateral anterior cruciate ligament reconstruction. Am J Sports Med. 2019;47(2):334–8. 10.1177/0363546518813901. (**in eng**). 30525891 10.1177/0363546518813901

[6] Nyland J, Moatshe G, Martin R. Combined ACL and anterolateral ligament reconstruction: time to pivot and shift the focus? Knee Surg Sports Traumatol Arthrosc. 2023;31(2):373–5. 10.1007/s00167-022-07072-6. (**in eng**). 35869981 10.1007/s00167-022-07072-6

[7] Wasdev A, P A, Krishnan R, Thomas A, M. GS, Amaravathi RS. Anatomical landmark technique for femoral tunnel placement of lateral extra-articular tenodesis. Arthrosc Tech. 2023;12(5):e779–86. 10.1016/j.eats.2023.02.007. (**in eng**). 37323791 10.1016/j.eats.2023.02.007 PMC10265728

[8] Mitrousias V, Chalatsis G, Komnos G, Neri T, Hantes M. Lateral augmentation procedures in anatomic anterior cruciate ligament reconstruction. How to avoid tunnel collision with intraoperative tunnel visualization: a technical note. Journal of ISAKOS. 2023;8(3):137–9. 10.1016/j.jisako.2023.03.001. (**in eng**). 36921765 10.1016/j.jisako.2023.03.001

[9] Tollefson LV, Shoemaker EP, Slette EL, Carlson M, LaPrade RF, Engebretsen L, et al. Adequate failure loads for modified Lemaire lateral extra-articular tenodesis are achieved with an interference screw, staple, and suture anchor: a biomechanical study of structural properties. Am J Sports Med. 2025;53(2):327–32. 10.1177/03635465241305739. (**in eng**). 39760525 10.1177/03635465241305739

[10] Abusleme S, Strömbäck L, Caracciolo G, Zamorano H, Cheyre J, Vergara F, et al. Lateral extra-articular tenodesis: a technique with an iliotibial band strand without implants. Arthrosc Tech. 2021;10(1):e85–9. 10.1016/j.eats.2020.09.029. (**in eng**). 33532213 10.1016/j.eats.2020.09.029 PMC7823113

[11] DePhillipo NN, Cinque ME, Chahla J, Geeslin AG, LaPrade RF. Anterolateral ligament reconstruction techniques, biomechanics, and clinical outcomes: a systematic review. Arthroscopy. 2017;33(8):1575–83. 10.1016/j.arthro.2017.03.009. (**in eng**). 28502387 10.1016/j.arthro.2017.03.009

[12] Pioger C, Gousopoulos L, Hopper GP, Vieira TD, Campos JP, El Helou A, et al. Clinical outcomes after combined ACL and anterolateral ligament reconstruction versus isolated ACL reconstruction with bone-patellar tendon-bone grafts: a matched-pair analysis of 2018 patients from the SANTI study group. Am J Sports Med. 2022;50(13):3493–501. 10.1177/03635465221128261. (**in eng**). 36255278 10.1177/03635465221128261

[13] Inderhaug E, Stephen JM, Williams A, Amis AA. Biomechanical comparison of anterolateral procedures combined with anterior cruciate ligament reconstruction. Am J Sports Med. 2017;45(2):347–54. 10.1177/0363546516681555. (**in eng**). 28027653 10.1177/0363546516681555

[14] Neri T, Dabirrahmani D, Beach A, Grasso S, Putnis S, Oshima T, et al. Different anterolateral procedures have variable impact on knee kinematics and stability when performed in combination with anterior cruciate ligament reconstruction. J ISAKOS. 2021;6(2):74–81. 10.1136/jisakos-2019-000360. (**in eng**). 33832980 10.1136/jisakos-2019-000360

[15] Grassi A, Olivieri Huerta RA, Lucidi GA, Agostinone P, Dal Fabbro G, Pagano A, et al. A lateral extra-articular procedure reduces the failure rate of revision anterior cruciate ligament reconstruction surgery without increasing complications: a systematic review and meta-analysis. Am J Sports Med. 2024;52(4):1098–108. 10.1177/03635465231173698. (**in eng**). 38294248 10.1177/03635465231173698 PMC10943615

[16] Ventura A, Legnani C, Boisio F, Borgo E, Peretti GM. The association of extra-articular tenodesis restores rotational stability more effectively compared to contralateral hamstring tendon autografts ACL reconstruction alone in patients undergoing ACL revision surgery. Orthop Traumatol Surg Res. 2021;107(2):102739. 10.1016/j.otsr.2020.06.022. (**in eng**). 33390331 10.1016/j.otsr.2020.06.022

[17] van der Wal WA, Meijer DT, Hoogeslag RAG, LaPrade RF. The iliotibial band is the main secondary stabilizer for anterolateral rotatory instability and both a Lemaire tenodesis and anterolateral ligament reconstruction can restore native knee kinematics in the anterior cruciate ligament reconstructed knee: a systematic review of biomechanical cadaveric studies. Arthroscopy. 2024;40(2):632-647.e1. 10.1016/j.arthro.2023.05.005. (**in eng**). 37207919 10.1016/j.arthro.2023.05.005

[18] Maffulli N. The early versus late anterior cruciate ligament reconstruction debate: history teaches us that we cannot use reason and evidence to fight and win against conviction. Arthroscopy. 2018;34(9):2524–5. 10.1016/j.arthro.2018.06.017. (**in eng**). 30173789 10.1016/j.arthro.2018.06.017

[19] Tanpowpong T, Kuptniratsaikul S, Itthipanichpong T, Limskul D, Thamrongskulsiri N, Supaluxmetha S, et al. Maximizing graft diameter: unleashing the power of triple-fold hamstring autografts for anterior cruciate ligament reconstruction. Arthrosc Tech. 2024;13(12):103131. 10.1016/j.eats.2024.103131. (**in eng**). 39780883 10.1016/j.eats.2024.103131 PMC11704893

[20] Rittweger J, Maffulli N, Maganaris CN, Narici MV. Reconstruction of the anterior cruciate ligament with a patella-tendon-bone graft may lead to a permanent loss of bone mineral content due to decreased patellar tendon stiffness. Med Hypotheses. 2005;64(6):1166–9. 10.1016/j.mehy.2004.06.037. (**in eng**). 15823709 10.1016/j.mehy.2004.06.037

[21] Jesani S, Getgood A. Modified Lemaire lateral extra-articular tenodesis augmentation of anterior cruciate ligament reconstruction. JBJS Essential Surg Tech. 2019. 10.2106/jbjs.St.19.00017. (**in eng**). 10.2106/JBJS.ST.19.00017 PMC6974308 32051777

[22] de Escuiro Oliveira D, Picchi Zaccharias V, Mayumi Horita M, Gabriel Betoni Guglielmetti L, Duarte Junior A, Baches Jorge P. Anterior cruciate and anterolateral ligament reconstruction using hamstring and peroneus longus tendons: surgical technique description. Arthrosc Tech. 2021;10(2):e397–402. 10.1016/j.eats.2020.10.030. (**in eng**). 33680771 10.1016/j.eats.2020.10.030 PMC7917200

[23] Giordano L, Maffulli N, Carimati G, Morenghi E, Volpi P. Increased time to surgery after anterior cruciate ligament tear in female patients results in greater risk of medial meniscus tear: a study of 489 female patients. Arthroscopy. 2023;39(3):613–22. 10.1016/j.arthro.2022.10.014. (**in eng**). 36309227 10.1016/j.arthro.2022.10.014

[24] Piedade SR, Leite Arruda BP, de Vasconcelos RA, Parker DA, Maffulli N. Rehabilitation following surgical reconstruction for anterior cruciate ligament insufficiency: what has changed since the 1960s?-state of the art. Journal of ISAKOS. 2023;8(3):153–62. 10.1016/j.jisako.2022.10.001. (**in eng**). 36410671 10.1016/j.jisako.2022.10.001

[25] El-Azab H, Moursy M, Mohamed MA, Elsayed M. A comparison of the outcomes of anterior curciate ligament reconstruction with large-size graft versus reconstruction with average-size graft combined with extraarticular tenodesis. Injury. 2023;54(3):976–82. 10.1016/j.injury.2023.01.033. (**in eng**). 36720663 10.1016/j.injury.2023.01.033

[26] Briggs KK, Lysholm J, Tegner Y, Rodkey WG, Kocher MS, Steadman JR. The reliability, validity, and responsiveness of the Lysholm score and Tegner activity scale for anterior cruciate ligament injuries of the knee: 25 years later. Am J Sports Med. 2009;37(5):890–7. 10.1177/0363546508330143. (**in eng**). 19261899 10.1177/0363546508330143

[27] Jakob RP, Stäubli HU, Deland JT. Grading the pivot shift. Objective tests with implications for treatment. J Bone Joint Surg Br. 1987;69(2):294–9. 10.1302/0301-620x.69b2.3818763. (**in eng**). 3818763 10.1302/0301-620X.69B2.3818763

[28] Gurtler RA, Stine R, Torg JS. Lachman test revisited. Contemp Orthop. 1990;20(2):145–54. 10148032

[29] Goncharov EN, Koval OA, Dubrov VE, Bezuglov EN, Filimonova AM, Goncharov NG. Clinical experience with combined reconstruction of the anterior cruciate and anterolateral ligaments of the knee in sportsmen. Int Orthop. 2019;43(12):2781–8. 10.1007/s00264-019-04409-8. 31511952 10.1007/s00264-019-04409-8

[30] Rosenstiel N, Praz C, Ouanezar H, Saithna A, Fournier Y, Hager JP, et al. Combined anterior cruciate and anterolateral ligament reconstruction in the professional athlete: clinical outcomes from the scientific anterior cruciate ligament network international study group in a series of 70 patients with a minimum follow-up of 2 years. Arthroscopy. 2019;35(3):885–92. 10.1016/j.arthro.2018.09.020. 30704884 10.1016/j.arthro.2018.09.020

[31] Ramjug S, Ghosh S, Walley G, Maffulli N. Isolated anterior cruciate ligament deficiency, knee scores and function. Acta Orthop Belg. 2008;74(5):643–51. 19058699

[32] Helito CP, Sobrado MF, da Moreira Silva AG, de Castro Pádua VB, Guimarães TM, Bonadio MB, et al. The addition of either an anterolateral ligament reconstruction or an iliotibial band tenodesis is associated with a lower failure rate after revision anterior cruciate ligament reconstruction: a retrospective comparative trial. Arthroscopy. 2023;39(2):308–19. 10.1016/j.arthro.2022.06.039. (**in eng**). 35840071 10.1016/j.arthro.2022.06.039

[33] Delaloye JR, Hartog C, Blatter S, Schläppi M, Müller D, Denzler D, et al. Anterolateral ligament reconstruction and modified Lemaire lateral extra-articular tenodesis similarly improve knee stability after anterior cruciate ligament reconstruction: a biomechanical study. Arthroscopy. 2020;36(7):1942–50. 10.1016/j.arthro.2020.03.027. 32251683 10.1016/j.arthro.2020.03.027

[34] Shybut TB. Editorial commentary: anterior cruciate ligament reconstruction alone is not sufficient in anterolateral complex injury: extra-articular augmentation (lateral extra-articular tenodesis [LET] or anterolateral ligament [ALL] reconstruction) allows surgeons to indicate tight (LET) or just right (ALL) on a case-by-case basis. Arthroscopy. 2022;38(3):925–7. 10.1016/j.arthro.2021.08.016. 35248237 10.1016/j.arthro.2021.08.016

[35] Migliorini F, Lucenti L, Mok YR, Bardazzi T, D’Ambrosi R, De Carli A, et al. Anterior cruciate ligament reconstruction using lateral extra-articular procedures: a systematic review. Medicina. 2025;61(2):294. 10.3390/medicina61020294. 40005410 10.3390/medicina61020294 PMC11857574

[36] Claes S, Vereecke E, Maes M, Victor J, Verdonk P, Bellemans J. Anatomy of the anterolateral ligament of the knee. J Anat. 2013;223(4):321–8. 10.1111/joa.12087. 23906341 10.1111/joa.12087 PMC3791125

[37] Ahn JH, Patel NA, Lin CC, Lee TQ. The anterolateral ligament of the knee joint: a review of the anatomy, biomechanics, and anterolateral ligament surgery. Knee Surg Related Res. 2019;31(1):12. 10.1186/s43019-019-0012-4. (**in eng**). 10.1186/s43019-019-0012-4 PMC7219606 32660576

[38] Daggett M, Stephenson C, Dobson J, Whitaker A, Redler A, Monaco E, et al. Anatomic and histological study of the anterolateral aspect of the knee: a SANTI group investigation. Orthop J Sports Med. 2018;6(10):2325967118799970. 10.1177/2325967118799970. 30345320 10.1177/2325967118799970 PMC6187433

[39] Helito CP, Demange MK, Bonadio MB, Tírico LE, Gobbi RG, Pécora JR, et al. Anatomy and histology of the knee anterolateral ligament. Orthop J Sports Med. 2013;1(7):2325967113513546. 10.1177/2325967113513546. 26535259 10.1177/2325967113513546 PMC4555517

[40] Hill JR, Eekhoff JD, Brophy RH, Lake SP. Elastic fibers in orthopedics: form and function in tendons and ligaments, clinical implications, and future directions. J Orthop Res. 2020;38(11):2305–17. 10.1002/jor.24695. 32293749 10.1002/jor.24695 PMC7572591

[41] Inderhaug E, Stephen JM, Williams A, Amis AA. Anterolateral Tenodesis or anterolateral ligament complex reconstruction: effect of flexion angle at graft fixation when combined with ACL reconstruction. Am J Sports Med. 2017;45(13):3089–97. 10.1177/0363546517724422. 28898106 10.1177/0363546517724422

[42] Sonnery-Cottet B, Lutz C, Daggett M, Dalmay F, Freychet B, Niglis L, et al. The involvement of the anterolateral ligament in rotational control of the knee. Am J Sports Med. 2016;44(5):1209–14. 10.1177/0363546515625282. 26865395 10.1177/0363546515625282

[43] Kittl C, El-Daou H, Athwal KK, Gupte CM, Weiler A, Williams A, et al. The role of the anterolateral structures and the ACL in controlling laxity of the intact and ACL-deficient Knee. Am J Sports Med. 2016;44(2):345–54. 10.1177/0363546515614312. 26657572 10.1177/0363546515614312

[44] Zappia M, Oliva F, Chianca V, Di Pietto F, Maffulli N. Sonographic evaluation of the anterolateral ligament of the knee: a cadaveric study. J Knee Surg. 2019;32(6):532–5. 10.1055/s-0038-1655763. 29852517 10.1055/s-0038-1655763

[45] Saithna A, Thaunat M, Delaloye JR, Ouanezar H, Fayard JM, Sonnery-Cottet B. Combined ACL and anterolateral ligament reconstruction. JBJS Essent Surg Tech. 2018;8(1):e2. 10.2106/jbjs.St.17.00045. (**in eng**). 30233974 10.2106/JBJS.ST.17.00045 PMC6143299

[46] Shashwat Agrawal, A. S. H., B. Seetharama Rao, Prajwal P. Mane, Chethan B. Shetty, Vikrant Khanna, Samarth Thakkar, Prajwal M. Divakar, Ankita Nigam, "Peroneus Longus Tendon Autograft for Primary Arthroscopic Reconstruction of the Anterior Cruciate Ligament," *Muscles, Ligaments and Tendons Journal,*10.32098/mltj.02.2023.08, 2023.

[47] Trinchese GF, Oliva F, Maffulli N. Minimally invasive anatomic reconstruction of the anterolateral ligament with ipsilateral gracilis tendon. Muscles Ligaments Tendons J. 2017;7(2):240–6. 10.11138/mltj/2017.7.2.240. (**in eng**). 29264334 10.11138/mltj/2017.7.2.240 PMC5725172

[48] Kannus P. “Structure of the tendon connective tissue,” in eng. Scand J Med Sci Sports. 2000;10(6):312–20. 10.1034/j.1600-0838.2000.010006312.x. 11085557 10.1034/j.1600-0838.2000.010006312.x

[49] Spencer L, Burkhart TA, Tran MN, Rezansoff AJ, Deo S, Caterine S, et al. Biomechanical analysis of simulated clinical testing and reconstruction of the anterolateral ligament of the knee. Am J Sports Med. 2015;43(9):2189–97. 10.1177/0363546515589166. 26093007 10.1177/0363546515589166

[50] Neri T, Cadman J, Beach A, Grasso S, Dabirrahmani D, Putnis S, et al. Lateral tenodesis procedures increase lateral compartment pressures more than anterolateral ligament reconstruction, when performed in combination with ACL reconstruction: a pilot biomechanical study. J isakos. 2021;6(2):66–73. 10.1136/jisakos-2019-000368. 33832979 10.1136/jisakos-2019-000368

[51] Schon JM, Moatshe G, Brady AW, Serra Cruz R, Chahla J, Dornan GJ, et al. Anatomic anterolateral ligament reconstruction of the knee leads to overconstraint at any fixation angle. Am J Sports Med. 2016;44(10):2546–56. 10.1177/0363546516652607. (**in eng**). 27407088 10.1177/0363546516652607

[52] Nazzal EM, Keeling LE, Ryan PM, Herman ZJ, Hughes JD. The role of lateral extra-articular tenodesis in anterior cruciate ligament reconstruction and treatment of rotatory knee instability: a scoping review. Curr Rev Musculoskelet Med. 2023;16(6):235–45. 10.1007/s12178-023-09832-4. 36995532 10.1007/s12178-023-09832-4 PMC10234940

[53] Kittl C, Halewood C, Stephen JM, Gupte CM, Weiler A, Williams A, et al. Length change patterns in the lateral extra-articular structures of the knee and related reconstructions. Am J Sports Med. 2015;43(2):354–62. 10.1177/0363546514560993. 25540293 10.1177/0363546514560993

[54] Kernkamp WA, Van de Velde SK, Hosseini A, Tsai TY, Li JS, van Arkel ERA, et al. In vivo anterolateral ligament length change in the healthy knee during functional activities-a combined magnetic resonance and dual fluoroscopic imaging analysis. Arthroscopy. 2017;33(1):133–9. 10.1016/j.arthro.2016.07.008. 27663034 10.1016/j.arthro.2016.07.008 PMC5179307

[55] Firth AD, Pritchett SL, Milner JS, Atkinson HF, Bryant DM, Holdsworth DW, et al. Quantitative magnetic resonance imaging of lateral compartment articular cartilage after lateral extra-articular tenodesis. Am J Sports Med. 2024;52(4):909–18. 10.1177/03635465241228193. 38385189 10.1177/03635465241228193

